# Novel Dietary Proteins Selectively Affect Intestinal Health In Vitro after *Clostridium difficile*-Secreted Toxin A Exposure

**DOI:** 10.3390/nu12092782

**Published:** 2020-09-11

**Authors:** Paulus G. M. Jochems, Johan Garssen, Pascale C. S. Rietveld, Coen Govers, Monic M. M. Tomassen, Harry J. Wichers, Jeroen van Bergenhenegouwen, Rosalinde Masereeuw

**Affiliations:** 1 Division of Pharmacology, Utrecht Institute for Pharmaceutical Sciences, Utrecht University, 3584 CG Utrecht, The Netherlands; p.g.m.jochems@uu.nl (P.G.M.J.); j.garssen@uu.nl (J.G.); p.c.s.rietveld@students.uu.nl (P.C.S.R.); b.j.vanbergenhenegouwen@uu.nl (J.v.B.); 2 Nutricia Research, Global Center of Excellence Immunology, 3584 CT Utrecht, The Netherlands; 3 Food & Biobased Research, Wageningen University & Research, 6708 WE Wageningen, The Netherlands; coen.govers@wur.nl (C.G.); monic.tomassen@wur.nl (M.M.M.T.); harry.wichers@wur.nl (H.J.W.)

**Keywords:** Caco-2, in vitro, intestine, clostridium difficile, dietary protein

## Abstract

Bacterial gastroenteritis forms a burden on a global scale, both socially and economically. The Gram-positive bacterium *Clostridium difficile* is an inducer of gastrointestinal bacterial infections, often triggered following disruption of the microbiota by broad-spectrum antibiotics to treat other conditions. The clinical manifestatiaons, e.g., diarrhea, are driven by its toxins secretion, toxin A (TcdA) and toxin B (TcdB). Current therapies are focused on discontinuing patient medication, including antibiotics. However, relapse rates upon therapy are high (20–25%). Here, eighteen dietary proteins were evaluated for their capacity to restore gut health upon *C. difficile*-derived TcdA exposure. We used bioengineered intestinal tubules to assess proteins for their beneficial effects by examining the epithelial barrier, cell viability, brush-border enzyme activity, IL-6 secretion, IL-8 secretion and nitric oxide (NO) levels upon TcdA challenge. TcdA effectively disrupted the epithelial barrier, increased mitochondrial activity, but did not affect alkaline phosphatase activity, IL-6, IL-8 and NO levels. Intervention with dietary proteins did not show a protective effect on epithelial barrier integrity or mitochondrial activity. However, bovine plasma and potato protein increased alkaline phosphatase activity, egg-white protein increased IL-6 and IL-8 release and wheat, lesser mealworm and yeast protein increased NO levels after TcdA exposure. Hence, dietary proteins can influence parameters involved in intestinal physiology and immune activation suggesting that supplementation with specific dietary proteins may be of benefit during *C. difficile* infections.

## 1. Introduction

Bacterial gastroenteritis is a common pathology worldwide, is associated with 1.5–2.5 million deaths per year and forms a great burden for society on a social and economic scale [1]. Clinical manifestations of gastrointestinal (GI) bacterial infections are vomiting, abdominal pain, nausea and diarrhea [1]. This can be induced by various mechanism such as mucosal invasion by pathogens and bacterial toxin production [1]. *Clostridium difficile* (*C. difficile*) is a Gram-positive bacterium and considered an enteric pathogen with global impact [2]. *C. Difficile* infections are a healthcare-associated problem and estimated to increase hospitalization costs by approximately $1.5 billion in the United States alone [3]. Many of the clinical manifestations are the result of the *C. difficile* release of toxin A and toxin B (TcdA and B) in the GI tract. TcdA and TcdB cause intestinal epithelial barrier disruption, resulting in an inflammatory environment leading to the loss of intestinal homeostasis. This process is associated with clinical manifestations, which range from mild diarrhea up to life-treating conditions [4,5,6,7]. *C. difficile* is an opportunistic bacterium causing infections during disrupted microbiota, often induced by broad-spectrum antibiotics [8]. The first step in treating a *C. difficile* infection is discontinuing any medications—mostly antibiotics—predisposing an individual to the infection. Thereafter, antibiotics are customized to the disease state, for which vancomycin or metronidazole are common initial therapies [5]. Unfortunately, these *C. difficile* therapies have high relapse rates (20–25%) and the antibiotic-resistant strains form a concern [6].

Alternative therapies using probiotic supplementation have been used in preventing *C. difficile* infections, however, clinical studies show ambiguous effects [2]. Hence, nutritional interventions are gaining interest as alternative to combat *C. difficile* infections. Functional foods are capable of inducing health benefits beyond their nutritional value and offer interesting candidates [9]. Proteins are often used as the backbone of complete nutritional interventions and are involved in almost all biologic processes within the human body [10]. In accordance, previous data suggested that food-derived proteins and/or protein fragments harbor functions that contribute to intestinal health, as reviewed by Martínez-Augustin et al. [11,12].

Here, we investigated the effects of novel protein sources for their capacity to recover TcdA-induced loss of intestinal integrity. We mimicked a *C. difficile* infection by exposing bioengineered intestinal tubules to TcdA, whereafter, animal- (whey, acidic whey, egg and blood plasma), plant- (soya, potato, pea, wheat and corn) and alternative- (insect, yeast and fungi) protein sources were evaluated for their recovery potential. To investigate beneficial effects on TcdA-induced inflammation, proteins were evaluated for their capacity to modulate cell viability, alkaline phosphatase activity, IL-6, IL-8 and nitric-oxide (NO) secretion.

## 2. Materials and Methods

### 2.1. Chemicals

All chemicals were purchased from Sigma-Aldrich (Zwijndrecht, The Netherlands) unless stated otherwise.

### 2.2. Caco-2 Cell Culture

Caco-2 cells (ATCC, Wesel, Germany) were maintained in high glucose Dulbecco’s Modified Eagle Medium (DMEM) (Gibco) supplemented with fetal calf serum (10% *v/v*) in the ThinCert^TM^ Transwells(Greiner Bio-One B.V., Alphen aan den Rijn, The Netherlands) model and additional penicillin and streptomycin (1% *v/v*) in the bioengineered intestinal tubules. Medium was refreshed every 2–3 days. Cells were passaged and seeded in the models when reaching 80–90% confluency.

### 2.3. Transwell^TM^ Model (TW)

Caco-2 cells were cultured in DMEM supplemented with 10% heat inactivated FBS (Hyclone Pervio, Ettenleur, The Netherlands). A total of 330,000 Caco-2 cells were seeded on ThinCert^TM^ Transwells with 33.6 mm^2^ membranes and 0.4-µm pores in 24-well culture plates. Cells were grown for 21 days at 5% CO_2_ and 37 °C and apical (150 µL) and basolateral (700 µL) media were replaced every 2–3 days.

### 2.4. Bioengineered Intestinal Tubules

The construction and cultivation of bioengineered intestinal tubules was described before [11]. In short, customized 3-dimensional polylactide (PLA) (Makerpoint, Utrecht, The Netherlands) chambers were printed and hollow fiber capillary membranes (HFMs) (SENUOFIL, Tianjin, China) were mounted in these chambers. After sterilization (30 min in 70% EtOH (*v/v*)), HFMs double coating with L-3,4-di-hydroxy-phenylalanine (5 h of 2 mg.mL^−1^ in 10-mM Tris buffer) followed by human collagen IV (2 h of 25 µg.mL^−1^ in PBS). Thereafter, 1.0 × 10^6^ Caco-2 cells were seeded onto HFM and incubated for 4 h. After seeding, devices were referred to as bioengineered intestinal tubules and cultivated for14 days under static condition and the final 7 days under flow shear stress. The latter was induced by putting the devices onto a 2-dimensional plate rocker. After 21 days of culturing, the bioengineered intestinal tubules were considered experiment ready.

### 2.5. Static In Vitro Digestion

Static in vitro digestion was performed according the widely accepted INFOGEST consensus with minor adaptations, as described before [11,13]. The pancreatin enzyme mixture was adjusted to a trypsin activity of 10 U/mL to assure compliance with cell cultures.

### 2.6. Toxin A and Potential Dietary Proteins

TW were coincubated with TcdA (0.25 µg/mL in apical culture medium) and one of the 18 protein digests (1:4 dilution in apical culture medium) for 6 h. Bioengineered intestinal tubules were exposed for 24 h to *C. difficile*-secreted TcdA (0.5 ug/mL in culture medium) (List Biologic Laboratories, Inc., Campbell, CA, USA) followed by 3-h exposure to one of the 18 potential dietary proteins digest (1:4 dilution in culture medium). Each experimental run included relevant control groups and different protein digests in-tandem. The potential dietary proteins had a variety of protein origin, animal, plant and alternative-based (see Table 1). More details with respect to the proteome and endotoxin contamination has previously been reported [11].

### 2.7. Transepithelial Electrical Resistance

To monitor the integrity of the Caco-2 monolayer in the TW, transepithelial electrical resistance (TEER) was measured at 37 °C using a Millicell-ERS Ω Meter (Millipore, Molsheim, France). TEER was determined before and 6 h after addition of a combination of TcdA and a protein digest. In each experiment, the digested compounds and control digest were exposed to Caco-2 cells in a technical duplicate and three biologic replicates were performed on different days. Results are shown relative to the TW model with culture medium exposure solely as control.

### 2.8. Inulin-FITC Leakage Assay

Paracellular permeability was quantified by inulin-FITC (0.1 mg.mL^−1^ in PBS or medium) perfusion of bioengineered intestinal tubules. This was done as described before [11]. In short, bioengineered intestinal tubules were washed with PBS and perfused with inulin-FITC for 10 min at a speed rate of 0.1 mL/min. Samples were taken from the chamber to assess inulin-FITC leakage by absorbance at excitation wavelength of 480–492 nm and emission wavelength of 518–520 nm using a multimode plate reader (Promega, Leiden, The Netherlands). Results are shown relative to positive control, unexposed bioengineered intestinal tubules. Bioengineered intestinal tubules were extracted from the 3D-chamber and cut in pieces and used for cell viability and alkaline phosphatase activity.

### 2.9. Immunofluorescent Staining and Zonula Occludens-1 Quantification

Immunofluorescent staining of the bioengineered intestinal tubules has been described before [14]. In short, cells were fixated (60% EtOH, 30% chloroform and 10% acetic acid (*v/v*)) for 5 min, permeabilized (0.3% (*v/v*) Triton X-100 in HBSS) for 10 min and blocked ((2% (*w/v*) bovine serium albumin (BSA) fraction V and 0.1% (*v/v*) Tween-20 in HBSS) for 30 min. Thereafter, bioengineered intestinal tubules were incubated with zonula occludens-1 (ZO-1) antibody (1:1000 diluted in blocking solution) (Thermo Fisher Scientific, Bleiswijk, The Netherland) for 2 h. After washing, a secondary antibody goat-anti-rabbit 594 (1:200) (Abcam, Cambridge, United Kingdom) was added. Finally, bioengineered intestinal tubules were mounted using Prolong gold-containing DAPI (Cell signaling technology, Leiden, The Netherlands) for nuclei staining. Images were acquired using the Leica TCS SP8 X (Leica Biosystems, Amsterdam, The Netherlands) confocal microscope. Immunofluorescent images are shown as maximum intensity projections of a z-stack.

### 2.10. Cell Viability

PrestoBlue^TM^ cell viability reagent assay (Thermo Fisher, Bleiswijk, The Netherlands) was used. PrestoBlue^TM^ reagent was mixed with culture medium at a 1:10 ratio and 100 µL was put on the bioengineered intestinal tubules in a 96-well plate. The plate was put in an incubator at 5% CO_2_ and 37 °C and incubated for 1 h protected from light. Thereafter, bioengineered intestinal tubules were removed and fluorescence was measured at excitation wavelength of 530 nm and emission wavelength of 590 nm using a Tecan infinite M200PRO plate reader (Tecan Austria GmbH, Grödig, Austria). An unexposed, but experiment-ready bioengineered intestinal tubule was used as positive control, which was set to 100% viability.

### 2.11. Alkaline Phosphatase Activity Assay

Bioengineered tubules were washed with PBS and alkaline phosphatase activity was measured using Amplite^TM^ Colorimetric Alkaline Phosphatase Assay kit (AAT Bioquest, Sunnyvale, CA, USA). The assay was performed according to the manufacturer’s protocol. In short, bioengineered intestinal tubule pieces were put in 50 µL PBS in a 96-well plate. Then, 50 µL of pNPP working solution was added to get a total volume of 100 µL. Thereafter, bioengineered intestinal tubules were incubated for 30 min protected from light at room temperature, where after absorbance was measured at 600 nm using a colorimetric plate reader (iMARK™ microplate absorbance reader, Bio-Rad, Veenendaal, The Netherlands). Values are shown relative to bioengineered tubules that were medium exposed.

### 2.12. IL-6 and IL-8 Secretion

After exposures, supernatant was collected and IL-8 (BioLegend, London, UK) and IL-6 (Biolegend, London, UK) were quantified by ELISA, executed according to the manufacturer’s protocol. First, plates were coated and incubated overnight. Thereafter, plates were blocked for 1 h and incubated with samples for 2 h, followed by detection-antibody incubation for 1 h and Avidin-HRP for 30 min. Wells were exposed for 15 min to substrate solution followed by stop solution. Absorbance was read at 450 nm. Wells were washed in between exposures. Values were corrected for background using the supernatant of the unseeded no-ECM-coated bioengineered intestinal tubules, specific for each data set.

### 2.13. Nitric Oxide Content

NO was determined via Griess reaction (Promega, Leiden, The Netherlands) according manufacturer protocol. In short, first samples were centrifuged for 3 min at 6000 rpm. Sulfanilamide solution was added to all wells and incubated for 10 min. Thereafter, NED solution was added and incubated for 10 min. Followed by an absorbance measurement at 490 nm.

### 2.14. Statistical Analysis

Statistical analysis was performed in GraphPad version 8 (GraphPad Software, San Diego, CA, USA), and data were analyzed for outliers using Grubbs test, α = 0.05. Thereafter, control groups (negative control, medium, TcdA and blank) were analyzed using a *t*-test whereas different dietary protein sources were compared to blank using one-way ANOVA followed by Dunnett’s test, considering a *p* value of <0.05 to be significant.

## 3. Results

Dietary proteins affect health parameters after *C. difficile-secreted* TcdA exposures. During a pilot study in the TW-model, 17 of the 18 protein sources were coincubated with TcdA and evaluated for their impact on barrier integrity. None of the potential proteins could prevent TcdA-induced barrier impairment (Figure S1 (Supplementary Materials)). To investigate their impact on intestinal epithelial health in more detail, these eighteen dietary protein sources were subdivided into animal-, plant- or alternative-based sources and evaluated for their recovery effect after TcdA-induced disruption. First, five animal-based protein sources (whey, acidic whey, egg white and bovine blood plasma) were evaluated for their capability to restore effects after TcdA exposure.

**Figure 1:**
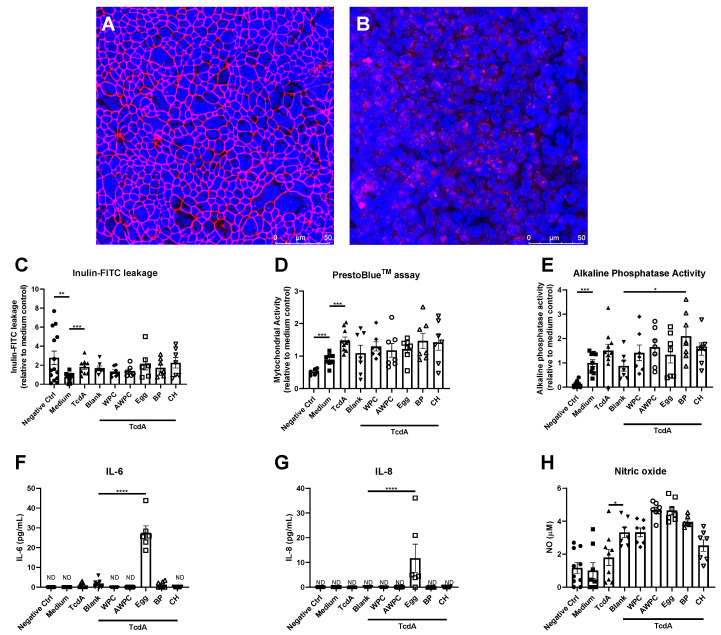
Potency of animal-based dietary protein to restore the effect of *clostridium difficile-secreted* toxin A (TcdA) on bioengineered intestinal tubules. (**A**) + (**B**) Representative images of bioengineered intestinal tubules (**A**) before and (**B**) after TcdA exposure showing nuclei in blue and zonula occludens-1 in red; (**C**–**H**) assessment of intestinal parameters after TcdA exposures, including (**C**) inulin-FITC leakage, (**D**) cell viability, (**E**) alkaline phosphatase activity, (**F**) release of IL-6, (**G**) IL-8 and (**H**) NO content. Data are presented as mean ± SD of at least 6 independent experiments and corrected for outliers (excluding 15 out of 424 data points). Control groups (negative control, medium, TcdA and blank) were tested for significance using a Student’s *t*-test. Protein sources were tested for significance using a one-way ANOVA followed by Dunnett’s test (compared to blank), * *p* < 0.05, ** *p* < 0.005, *** *p* < 0.001 and **** *p* < 0.0001. ND, not detectable.

Experiment-ready bioengineered intestinal tubules were viable and demonstrated to have a functional epithelial barrier including a clear ZO-1 network and increased alkaline phosphatase activity compared to the negative control (Figure 1A,C–E). TcdA exposure resulted in a disruption of the intestinal epithelial barrier as observed by an increase in inulin-FITC leakage (Figure 1C) and disrupted ZO-1 network in the bioengineered intestinal tubules (Figure 1B). Mitochondrial activity, commonly used as cell viability marker, was increased upon TcdA exposure (Figure 1D). Alkaline phosphatase activity, IL-6, IL-8 and NO were not affected by TcdA (Figure 1E–H). To correct for the effect of the digestive enzymes present in the protein digest, experiment-ready bioengineered intestinal tubules were exposed to medium containing only digestive enzymes (blank). The digestive enzymes had no effect on epithelial integrity, cell viability, brush-border enzyme activity, IL-6 and IL-8 (Figure 1C–G). However, it did increase NO content in comparison to the TcdA exposed group (Figure 1H). Comparing the five animal-based protein sources to blank, did not reveal restorative effects on the intestinal epithelial integrity (Figure 1C), mitochondrial activity (Figure 1D) or NO content (Figure 1H) after TcdA exposure. However, bovine plasma (BP) increased alkaline phosphatase activity (Figure 1E) and egg-white protein (egg) increased IL-6 and IL-8 secretion (Figure 1F–G).

Next, seven plant-based protein sources (soya bean, pea, wheat, potato and corn) were evaluated on their capability to induce beneficial effects after TcdA exposure.

**Figure 2:**
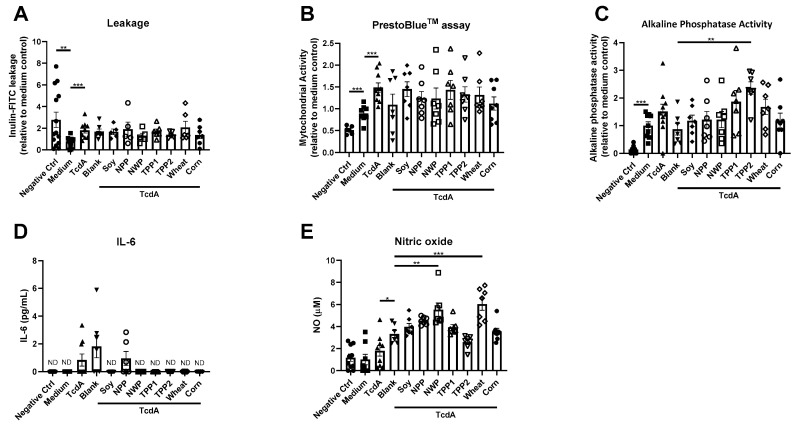
Potency of plant-based dietary protein to restore the effect of *Clostridium difficile-secreted* toxin A (TcdA) on bioengineered intestinal tubules. (**A**–**E**) Assessment of intestinal parameters after TcdA exposures, including (**A**) inulin-FITC leakage, (**B**) cell viability, (**C**) alkaline phosphatase activity, (**D**) release of IL-6 and (**E**) NO content. Data are presented as mean ± SD of at least a 6 independent experiments and corrected for outliers (excluding 24 out of 431 data points). Control groups (negative control, medium, TcdA and blank) were tested for significance using a Student’s *t*-test. Protein sources were tested for significance using one-way ANOVA followed by Dunnett’s test (compared to blank), * *p* < 0.05, ** *p* < 0.005, and *** *p* < 0.001. ND, not detectable.

Comparing the seven plant-based protein sources to blank control, did not lead to recovery of the intestinal epithelial integrity (Figure 2A), mitochondrial activity (Figure 2B) or IL-6 (Figure 2D) after TcdA exposure. IL-8 was below the limit of detection for all plant-based dietary proteins and was, therefore, not shown. However, potato protein, TPP2, increased alkaline phosphatase activity (Figure 2C). In turn, wheat protein sources (NWP and wheat), increased NO production in the bioengineered intestinal tubules (Figure 2E).

The third series of experiments consisted of alternative-based dietary proteins originating from insect, yeast and fungi, that were evaluated for their restoring capabilities after *Clostridium difficile-secreted* TcdA exposure.

**Figure 3:**
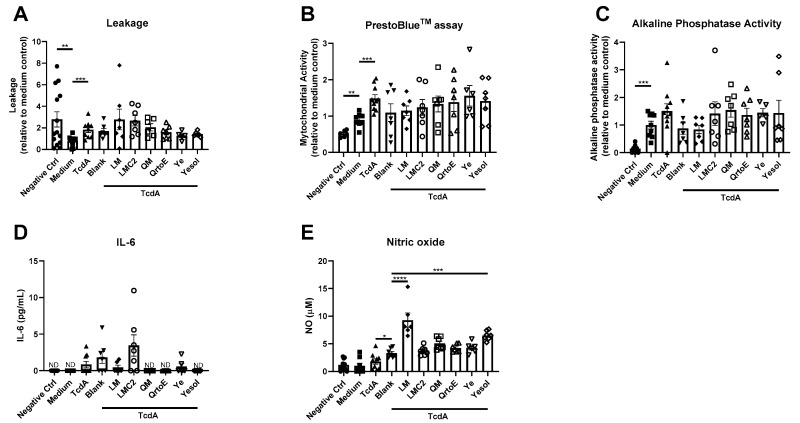
Potency of alternative-based dietary protein to restore the effect of *Clostridium difficile-secreted* toxin A (TcdA) on bioengineered intestinal tubules. (**A**–**E**) Assessment of intestinal parameters after TcdA exposures, including (**A**) inulin-FITC leakage, (**B**) cell viability, (**C**) alkaline phosphatase activity, (**D**) release of IL-6 and (**E**) NO content. Data are presented as mean ± SD of at least a 6 independent experiments and corrected for outliers (excluding 15 out of 386 data points). Control groups (negative control, medium, TcdA and Blank) were tested for significance using a Student’s t-test. Protein sources were tested for significance using one-way ANOVA followed by Dunnett’s test (compared to blank), * *p* < 0.05, ** *p* < 0.005, *** *p* < 0.001 and **** *p* < 0.0001. ND, not detectable.

Comparing six alternative-based protein sources to blank control, revealed no reparative effects on the intestinal epithelial integrity (Figure 3A), mitochondrial activity (Figure 3B), alkaline phosphatase activity (Figure 3C) or IL-6 (Figure 3D) after TcdA exposure. IL-8 was below the limit of detection for all alternative-based dietary proteins and was, therefore, not shown. However, alternative protein sources originating from insect (LM) and yeast (YE), increased NO content (Figure 3E).

## 4. Discussion

In this study, we evaluated the potential beneficial effects of protein supplementation of *C. difficile*-derived TcdA-induced negative effects on gut health, which may eventually be of importance for relieving symptoms for various intestinal diseases e.g., IBD or food allergy. Proteins were obtained from different sources and suppliers and were partially characterized earlier [11]. A limitation of our study was that potential impurities of the proteins that may have had a biologic activity were not analyzed. Preclinical screening for bioactivity often occurs by using in vitro models with moderate-to-high throughput, along with cost and ethical advantages over animal and clinical experimentations. Cultivation of Caco-2 cells on Transwell^TM^ inserts is considered the gold standard for in vitro intestinal-like epithelial barrier assessment, however, with some limitations [15]. For example, Caco-2 monolayers on Transwell^TM^ inserts lack in vivo-like morphology and show limited differentiation [16]. These drawbacks have been addressed by the development of bioengineered intestinal tubules with improved physiological aspects such as the presence of the main epithelial cell types (e.g., absorptive enterocytes, goblet cells, Paneth cells, enteroendocrine cells and stem cells) and the occurrence of villi-like structures [14]. Here, we showed that experiment-ready bioengineered intestinal tubules are comprised of a viable and functional epithelial monolayer expressing brush-border enzyme activity. Demonstrated by the presence of alkaline phosphatase activity, ZO-1 expression and leak-tightness. Although bioengineered intestinal tubules show improvements over the currently often applied Caco-2 Transwell^TM^ model, in general in vitro models remain a simplified representation of the in vivo situation. Eventually, a combination of experimental data with computational approaches (i.e., in silico modeling) may complement the system and further advance the extrapolation towards in vivo.

We previously demonstrated the sensitivity of the bioengineered intestinal tubules to TcdA exposure, resulting in an increased inulin-FITC leakage [14]. The disruptive effect of TcdA on the epithelial barrier in the bioengineered intestinal tubules is here confirmed and in line with previous research. Loss of barrier function exposes the underlying cells to luminal products. This could be a critical step in the initiation of the immune response. If the barrier function can be protected, there is reason to assume subsequent inflammatory responses can be ameliorated or inhibited [17]. In this work, we analyzed both the function of the barrier as well as the epithelial stress response measured as cytokine release. Although epithelial cytokine (and other mediators) release is important to initiate and maintain the immune response, it is currently unclear how much they would contribute to the immune response provoked by immune cells exposed to luminal content. Since our interventions may impact either gut function and/or epithelial-soluble mediator release we aimed to measure both aspects in context of recovery. In vivo maintenance of a viable and functional epithelial monolayer, including brush-border enzyme activity, is essential for mucosal homeostasis [18]. Loss of intestinal barrier integrity leads to translocation of bacteria and bacterial products to the lumen provoking local and potentially systemic inflammatory responses [19]. TcdA itself has, next to its barrier disruptive effect, a wide range of biologic effects, e.g., induction of cytotoxicity and release of the proinflammatory cytokine, IL-8 [20,21]. According to this knowledge, parameters of interest were expanded considering cell viability and IL-8 content and additionally the effect on alkaline phosphatase, IL-6 and NO of which little is known after TcdA exposure. Our data show that TcdA did not affect alkaline phosphatase, IL-6, IL-8 and NO content. Although the upregulation of IL-8 has been reported upon TcdA exposure, this was based on mRNA expression solely [21]. Here, secretion of IL-8 protein rather than IL-8 expression was taken into account. A possible explanation for this discrepancy may be that increases in gene expressions not always lead to increases in protein expressions due to a variety of biologic processes, such as efficiency between transcripts, protein turnover and cellular stress [22]. Further, the current model used does not allow investigating the role of the immune system in driving the intestinal cytokine responses following TcdA exposure. To have a better understanding of the potential beneficial immunomodulatory effects of protein supplementation on the TcdA-induced disruptive effect, the model should be further developed and complemented with immune cells.

Mitochondrial activity was increased upon TcdA exposure, known to disrupt mitochondrial homeostasis [23]. Previously, it was shown that exposure of HT29 epithelial cells to TcdA resulted in an increase ROS production. Increased ROS levels were associated with mitochondrial dysfunction which could lead to loss of mitochondrial activity [24]. Our data did not show TcdA-induced changes in NO and NO itself is not the primary mitochondrial ROS and is known to decrease mitochondrial electron carrier capacity [25,26]. However, Prestoblue^TM^ measurements could also point towards increased cell proliferation, indicating that future studies should be aimed at mechanistically investigating TcdA effects on mitochondrial function [27].

Neither animal-, plant- nor alternative-based proteins were able to restore TcdA-induced loss of barrier integrity, based on inulin-FITC leakage in the bioengineered intestinal tubules nor in the TW. In addition, none of the dietary proteins were able to restore TcdA-induced increases in the Prestoblue^TM^ assay. Caco-2 cell monolayers are associated with differentiation rather than proliferation, and TcdA monolayer disruption may therefore influence the mitochondrial state of the Caco-2 cells [28]. Hence, mitochondrial dysfunction has been associated with a loss in epithelial barrier itself and forms an interesting target [29]. For example polyamines have shown to be capable of increasing mitochondrial activity in the intestine, underscoring the potential of dietary proteins to alter the mitochondrial activity [30]. On the other hand, alkaline phosphatase activity, IL-6, IL-8 and NO content were affected by some protein digests after TcdA. Alkaline phosphatase is one of many brush-border enzymes contributing to the absorptive process of lipids right before absorption [31]. More interesting is its capacity to dephosphorylate the active/toxic Lipid A moiety of endotoxins, a major component of the Gram-negative bacteria cell wall, resulting in detoxification of active endotoxins [19]. Endotoxins are not capable of penetrating across a healthy intestinal epithelium [32]. However, during a state of the intestinal epithelial impairment, e.g., during a *C. difficile* infection, there is an increased risk of diffusion of endotoxins through the epithelial barrier triggering a systemic inflammatory response [33]. Moreover, alkaline phosphatase is involved in other physiological functions such as regulation of gut microbial communities by promoting commensal organism colonization [34,35]. Intestinal alkaline phosphatase has been used effectively as a therapeutic agent in a murine animal model against *C. difficile*-associated diseases and induction of alkaline phosphatase activity has shown merit in the prevention of inflammatory diseases, e.g., inflammatory bowel disease [36,37,38]. Here BP, an animal-based protein from bovine plasma and TPP2, a plant-based protein from potato, increased the alkaline phosphatase activity after TcdA exposure. Diet is known to modulate alkaline phosphatase activity, for example L-cysteine and L-phenylalanine are linked to alkaline phosphatase activity inhibition [39,40]. This is in line with the results observed for the potato protein, which is low in cysteine content and could be the reason for its inability to affect alkaline phosphatase activity [41]. Another dietary component, zinc, is reported to increase intestinal alkaline phosphatase activity [42]. Zinc is one of the common microelements in bovine blood plasma could, therefore, be the reason for the increase in alkaline phosphatase activity observed after BP [43]. However, whether or not zinc is present in our BP isolate remains to be determined.

Intestinal epithelial cells are involved in orchestrating the immune system via cyto- and chemokine release [44]. After TcdA exposure, egg-white protein increased IL-6 and IL-8 secretion. These cytokines are increased during an inflammatory state and are involved in T-helper cell differentiation and neutrophil recruitment, respectively [45,46]. Egg protein contains a variety of bioactive compounds with both anti- (e.g., lutein) and proinflammatory (e.g., cholesterol) capacities [47]. Although contradictory findings have been reported, overall findings argue for proinflammatory effects within healthy individuals and anti-inflammatory effects in individuals suffering from metabolic syndrome or type-2-diabetes [48,49,50,51]. Furthermore, plant- and alternative-based proteins (NWP, wheat, LM and Yesol) increase NO content after TcdA exposure. It has been shown before that *C. difficile* consumes nitrate and nitrite without producing the ROS NO, and TcdA-induced cytotoxicity in macrophages is not NO-mediated [52,53]. This is supported by our data as TcdA exposure did not increase NO content. Altogether, these results are indicative for a limited role of NO during *C. difficile* infections. However, the physiological role of NO can possibly benefit individuals suffering from *C. difficile* infections as an increased NO content is associated with decreased intestinal motility [54]. *C. difficile* infections are associated with diarrhea, which in turn is associated with an increased motility [55]. Hence, the increased NO levels may find use in counter playing this clinical manifestation. Further research should be directed whether these levels of NO have a biologic effect on motility.

## 5. Conclusions

In conclusion, exposure of epithelial barriers to *C. difficile*-derived TcdA resulted in an epithelial barrier disruptive effects in TW and in bioengineered intestinal tubules. Eighteen protein digests were investigated for their potential beneficial effect. From these, seven digests showed promising data. BP and TPP2 increased alkaline phosphatase activity, thought to be beneficial due to its endotoxin detoxifying capacity. Egg resulted in a more proinflammatory environment as it increased IL-6 and IL-8, which could enhance immune surveillance. Finally, NWP, wheat, LM and Yesol increased NO, which can decrease intestinal motility, possibly counter acting the clinical manifestation of diarrhea. Based on this data set, further research into protein digest, potentially as a component of dietary intervention, is promising, but more research is needed before treatment of *C. difficile* and its associated negative health effects by dietary proteins can be taken into consideration.

## Figures and Tables

**Table 1:** Overview of the 18 dietary proteins evaluated, including their characteristics and distributors.

Full Name	Protein Source	Abbreviation	Protein Origin	Carbohydrates (%)	Fat (%)	Protein (%)	Kcal/100 g	Distributor
Whey protein concentrate	Whey	WPC	Animal	17	0.4	74	N/A	Danone Nutricia Research
Acidic whey protein concentrate	Whey	AWPC	Animal	7	8	82	N/A	Danone Nutricia Research
Egg	Egg white	Egg	Animal	4.5	0.3	82.2	373	Bulk Powders
Soy	Soya Bean	Soy	Plant	1	3.3	91	378	Bulk Powders
NPP	Pea	NPP	Plant	3	6	80	395	Roquette
NWP	Wheat	NWP	Plant	4	7	82	409	Roquette
Bovine plasma	Blood protein	BP	Animal	0	2	72	298	Darling Ingredients
Collagen hydrolysate	Blood protein	CH	Animal	0	0	91.8	360	Darling Ingredients
Lesser mealworm	Insect	LM	Alternative	11	30.9	58	N/A	Proti-Farm
Lesser mealworm concentrate 2	Insect	LMC2	Alternative	N/A	N/A	N/A	N/A	Proti-Farm
TPP1	Potato	TPP1	Plant	0	5	95	378	Avebe
TPP2	Potato	TPP2	Plant	2	0	90	374	Avebe
Quorn mycoprotein	Fungi	QM	Alternative	5.6	7.5	53	361	Quorn Foods
Quorn ready-to-eat	Fungi/egg	QrtoE	Alternative	1.7	2.6	13.8	417	Quorn Foods
Wheat protein	Wheat	Wheat	Plant	9	5	79	399	Cargill
Corn protein	Corn	Corn	Plant	4	0.4	84	370	Cargill
Yeast	Yeast	YE	Alternative	1.2	3.2	88.4	N/A	Lesaffre
Yesol	Yeast	Yesol	Alternative	N/A	N/A	N/A	N/A	Lesaffre

N/A—not available.

## Supplementary Materials

The following are available online at https://www.mdpi.com/2072-6643/12/9/2782/s1, Figure S1: Pilot study in Transwell^TM^ inserts show no protective effect against TcdA-induced epithelial disruption.
