# Supplementary material for: Novel Dietary Proteins Selectively Affect Intestinal Health In Vitro after Clostridium difficile-Secreted Toxin A Exposure
Source: Nutrients. 2020 Sep 11;12(9):2782. doi: 10.3390/nu12092782 (PMC7551268; doi:10.3390/nu12092782)
Supplement: Supplementary file 1 [file nutrients-12-02782-s001.pdf]

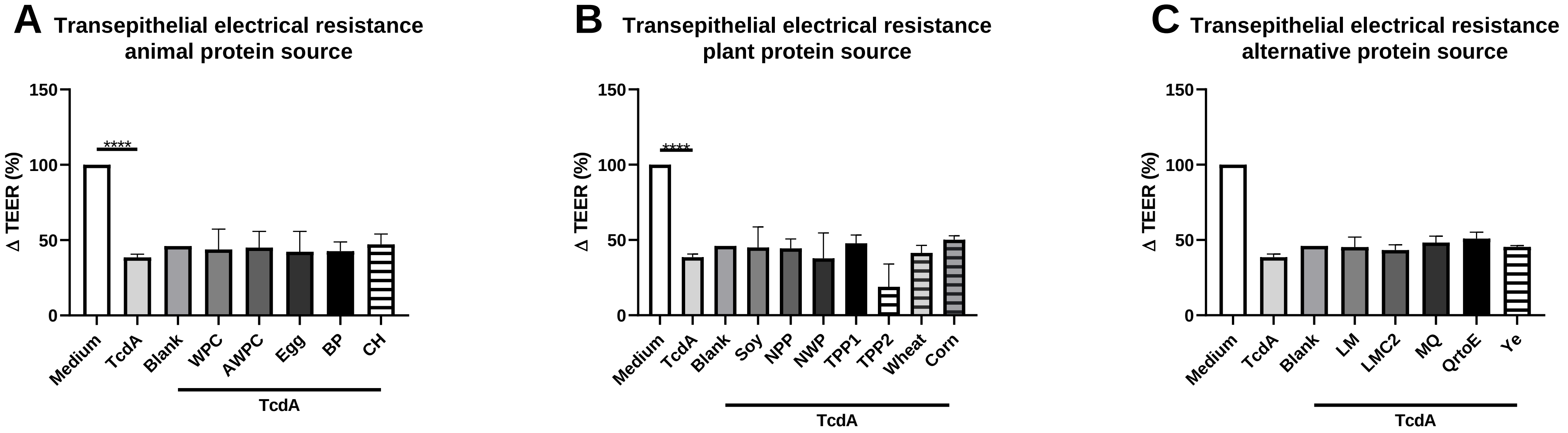
A
Transepithelial electrical resistance
animal protein source
150
100
50
0
Δ TEER (%)
****
Medium
TcdA
Blank
WPC
AWPC
Egg
BP
CH
TcdA
B
Transepithelial electrical resistance
plant protein source
150
100
50
0
Δ TEER (%)
****
Medium
TcdA
Blank
Soy
NPP
NWP
TPP1
TPP2
Wheat
Corn
TcdA
C
Transepithelial electrical resistance
alternative protein source
150
100
50
0
Δ TEER (%)
Medium
TcdA
Blank
LM
LMC2
MQ
QrtoE
Ye
TcdA
